# Exploring Maternal Mental Health Conditions and Urban-Rural Disparities in Hypertensive Disorders of Pregnancy in South Carolina: A Fairlie Decomposition Approach

**DOI:** 10.1007/s10995-026-04239-2

**Published:** 2026-02-18

**Authors:** Manan Roy, Maggie Sugg, Jennifer Runkle

**Affiliations:** 1 Department of Nutrition and Health Care Management, Appalachian State University, Boone, NC 28608 USA; 2 Department of Geography and Planning, Appalachian State University, Boone, NC 28608 USA; 3 North Carolina Institute for Climate Studies, North Carolina State University, Asheville, NC 28803 USA

**Keywords:** Maternal mental health, Hypertensive disorders of pregnancy, Decomposition, Urban rural disparity

## Abstract

**Objectives:**

Reproductive complications vary across rural and urban areas in the Southeastern U.S., yet maternal mental health–an important precursor to such complications–remains understudied. This study is the first to examine how mental disorders may contribute to disparities in hypertensive disorders of pregnancy (HDP) between urban and rural settings.

**Methods:**

We used South Carolina (SC) data on delivery hospitalizations for more than 270,000 mothers presenting with HDP who have given birth only once between 1999 and 2017. A Fairlie decomposition method was performed to examine the contribution of diagnosed maternal mental health conditions, and social inequalities account for the urban-rural differences in HDP prevalence.

**Results:**

The prevalence of HDP was 5.75% among urban mothers and 6.16% among rural mothers. Fairlie decomposition results indicated that maternal factors such as education attainment, chronic hypertension, and gestation age explained 90% of the geographic disparities in the HDP prevalence. Interestingly, differences in maternal mental health conditions, such as mental disorders of pregnancy and perinatal mood and anxiety disorders, reduced the urban-rural gap in the HDP prevalence.

**Conclusions for Practice:**

Our results highlight the importance of individual and community drivers required to improve maternal mental health conditions. More community support systems for the mental health along with reproductive health will greatly improve maternal health outcomes.

## Significance

What is Already Known on this Subject?

What this Study Adds?

HDP diagnoses tend to be more common among rural individuals, the potential mental health effects of HDP, and the well-documented birth complications and subsequent hypertension complications following HDP.

The link between mental health conditions and HDP have yet to be analyzed. To the best of our knowledge, this study is the first to quantify the role of diagnosed maternal mental health experiences in analyzing the rural-urban differences in the probability of an HDP diagnosis.

## Introduction

Hypertensive disorders of pregnancy (HDP) are among the leading causes of maternal and fetal morbidity and mortality worldwide and pose a critical threat to maternal and infant health (Garovic et al., 2020). In 2013, the American Congress of Obstetricians and Gynecologists guidelines classified HDP into four categories: gestational hypertension, preeclampsia/eclampsia, chronic hypertension, and chronic hypertension complicated with preeclampsia/eclampsia (Obstet Gynecol. 2013). Since HDP is potentially associated with cardiovascular disease, the 2020 International Society of Hypertension guidelines emphasize the importance of blood pressure monitoring during pregnancy (Unger et al., 2020).

Recent evidence has shown an increase in the incidence rate of HDP over the past few decades (Auger et al., 2016; Rezende et al., 2016) suggesting that HDP will become increasingly important in the future (Ananth et al., 2013).In the United States (U.S.), the incidence of new-onset HDP doubled from 2007 to 2019, with persistent rural‐urban differences across the United States (Cameron et al., 2022). New‐onset HDP is defined as an incident hypertension diagnosis arising during pregnancy (gestational hypertension and preeclampsia/eclampsia). This condition is associated with more than double the risk of coronary heart disease, heart failure, stroke, and cardiovascular‐related mortality (Grandi et al., 2019; Wu et al., 2017) and accounts for up to 44% of maternal deaths in the U.S. within the first six days postpartum (Petersen et al., 2019).

Highlighting social determinants of health that affect HDP is an important step toward improving maternal cardiovascular health. Specifically, individuals in rural areas face distinct challenges such as greater distance to care, lower access to care and high-risk facilities, that play a role in the rural-urban gap in adverse pregnancy outcomes (Harrington et al., 2020; Obstet Gynecol. 2014). From 2007 to 2019, per 1000 live births, the age‐adjusted incidence of HDP increased among both rural and urban pregnant women, the incidence increased from 48.6 (95% confidence interval (CI), 48.0–49.2) to 83.9 (95% CI, 83.1–84.7) in rural areas and from 37.0 (95% CI, 36.8–37.2) to 77.2 (95% CI, 76.8–77.6) in urban areas. However, the rate of annual increase in new‐onset HDP was more rapid after 2014, with greater acceleration in urban areas relative to rural areas. Rate ratios (95% CI) comparing the incidence of new‐onset HDP in rural and urban areas thus declined from 1.31 (1.30–1.33) in 2007 to 1.09 (1.08–1.10) in 2019. (Cameron et al., 2022).

At the same time, Runkle et al. (Runkle et al., 2023) note that the identification and treatment of mental health issues in pregnant individuals are crucial in preventing adverse maternal and neonatal outcomes, including HDP. For pregnant individuals, poor mental health can increase the likelihood of preterm labor, postpartum mental health issues, preeclampsia, hypertension, diabetes, suicide, and infanticide (Cox et al., 2016; Franks et al., 2017; Watson et al., 2019). Additionally, untreated maternal mental health conditions cost the United States healthcare system upwards of $15 billion dollars every year (Cox et al., 2016).

Although psychosocial factors have been implicated as both a cause and consequence of hypertension in the general population, the link is less understood in the case of HDP (Shay et al., 2020). Recent research has linked maternal mental health during the preconception, prenatal, and postpartum periods to increased risk for leading maternal obstetric complications, such as HDP (Runkle et al., 2023; Montagnoli et al., 2020). A systematic review and meta-analysis of 44 studies encompassing 61.2 million pregnancies found that depression and/or anxiety diagnosed prior to measurement of hypertension was statistically associated with a new hypertensive disorder during pregnancy, and individuals experiencing depression or anxiety during pregnancy have an increased prevalence of HDP compared to their non-depressed or non-anxious counterparts (Shay et al., 2020).

Despite the vast literature establishing that HDP diagnoses tend to be more common among rural individuals, the potential mental health effects of HDP, and the well-documented birth complications and subsequent hypertension complications following HDP, the link between mental health conditions and HDP has yet to be analyzed. Therefore, our analysis is guided by two research questions: (1) Does the likelihood of being diagnosed with HDP vary based on the mother’s rurality status - urban versus rural? (2) How are these differences explained by clinical factors, especially diagnosed mental health conditions and other individual sociodemographic factors?

To our knowledge, this study is the first to quantify the role of diagnosed maternal mental health experiences in analyzing the rural-urban differences in the probability of an HDP diagnosis. This research is timely and critical for U.S. policy development. For example, the Surgeon General’s Call to Action to improve maternal health notes mental health conditions are also common complications during pregnancy and in the postpartum period and may contribute to poor maternal outcomes. The Call to Action cites data from 14 state MMRCs between 2008 and 2017 showing that almost 10% of pregnancy-related deaths were due (in whole or in part) to mental health conditions. (USDHHS 2020) Our data and analytical strategy allow us to address this gap by highlighting the contribution of individual and community drivers to maternal mental health conditions.

## Data and Measurement

### South Carolina Hospital Discharge Data

To examine the role of maternal mental health experiences in explaining the urban-rural gap in the probability of an HDP diagnosis, we used hospital delivery discharge data obtained from the South Carolina (SC) Revenue and Fiscal Affairs Office Health and Demographics division for all hospital labor and deliveries in South Carolina from 1999 to 2017. Hospital delivery discharges were linked with infant birth and death records using a unique maternal identification number. Only delivery hospitalizations for pregnant women with a residence in South Carolina, with a gestational age of 20 weeks or more, and age 18–50 years were included in the initial sample. The study authors’ University’s Institutional Review Board deemed this research exempt (IRB#19–0272).

### Analysis Sample

Despite the richness of the SC longitudinal dataset on delivery hospitalizations, following Puro et al. (Puro et al., 2022) to reduce the heterogeneity of the sample and to account for the confounding relationship between the history of clinically complex pregnancies and mental health experiences, the analysis sample comprised pregnant women who have given birth only once in South Carolina from 1999 to 2017. A total of 278,723 pregnant women were included in our sample. Among these pregnant women, 228,420 (81.95%) lived in urban areas, and 50,303 (18.05%) lived in rural areas. The HDP prevalence rate areas among rural pregnant women is 6.1% (3,099 of 50,303) while the urban HDP prevalence rate is 5.7% (13,156 of 228,420).

### Variable Definitions

#### Dependent Variable: Hypertensive Disorders of Pregnancy

The primary dependent variable was a binary indicator of whether a mother was diagnosed with HDP, coded using the International Classification of Diseases, Ninth Revision (ICD-9) codes 642.3, 642.4, 642.5, 642.6, 642.7.

#### Main Independent Variables: Maternal Mental Health Disorders

The main independent variables of interest were the mental health disorders of pregnant women. Borrowing from Runkle et al. (Runkle et al., 2023) and McKee et al. (McKee et al., 2020), hospital delivery records at discharge were used to define the following outcomes based on ICD9: (1) perinatal mood and anxiety disorders (PMAD) and (2) maternal mental disorders of pregnancy (MDP). MDP is a diagnosis used to document any mental disorder complicating pregnancy, childbirth, or the puerperium (Kelly et al., 2001). PMAD is a specific diagnosis code to record mood or anxiety disorders during pregnancy and up to one year after delivery. For this analysis, we operationalize perinatal mental health to include (1) all mental disorders that preexist or recurred during gestation and incident conditions that emerged during the prenatal or postpartum periods (i.e., PMAD) and (2) any mental disorder that first emerged during pregnancy (i.e., MDP) (Montagnoli et al., 2020).

#### Other Covariates

Covariates included the following maternal factors: age in years, educational attainment (a high school degree, some college, college, and more than the highest level of college such as Bachelor’s degree), race and ethnicity (non-Hispanic White, non-Hispanic Black, Hispanic, and Other (i.e., Asian,…), insurance status (uninsured, Medicare/Medicaid private, U.S. Veteran Affairs (VA), Kotelchuk prenatal care adequacy index (inadequate, intermediate, adequate, and adequate plus) (Kotelchuck, 1994), gestational age (weeks), history of diabetes (yes/no), chronic hypertension (yes/no), obesity (yes/no), and gestational diabetes (yes/no). The year of discharge from the delivery hospitalization was also controlled for in the analysis. Rural compared to an urban maternal residence was coded using 2010 rural-urban community area codes (RUCA) to zip code areas by relying on the 5-tiered coding schema. By this definition, RUCA codes 1–6 denote urban areas, and 7–10 denote rural areas. Due to data restrictions, data on hospitals and providers was unavailable.

## Methods

### Descriptive Statistics

The chi-square test was used to anlyze the distribution characteristics of categorical variables related to the SC pregnant women in urban and rural areas. The t-test was used to analyze the difference in means of continuous variables related to SC pregnant women in urban and rural areas. Significance was defined as α = 0.05.

### Logistic Models

For rural and urban samples, two separate logistic models were estimated to explore the association of HDP with PMAD only and with MDP only. All models controlled for other important individual sociodemographic factors. An odds ratio (OR) > 1 indicated a risk factor for HDP, and pregnant women with these types of characteristics were more inclined to have a higher likelihood of HDP; conversely, an OR < 1 indicated a factor promoting lower HDP, and pregnant women with these types of characteristics were more inclined to have a lower probability of HDP. Stata 18 was used for statistical analysis (StataCorp, 2023).

### Fairlie Decomposition

The Fairlie model was adopted to decompose the causes and related contributors to the rural/urban disparity in the prevalence of HDP. The feasibility of the Fairlie model in health inequalities decomposition is verified by several studies (Yuan et al., 2023).

First, the binary logistic regression models of the urban and rural pregnant women’s HDP status were established as follows:

$\:{Y}^{a}=F\left({X}^{a}{\beta\:}^{a}\right)$ and $\:{Y}^{b}=F\left({X}^{b}{\beta\:}^{b}\right)$ where *F* is the cumulative distribution function from the logistic distribution for HDP diagnosis (*Y*).

$\:{Y}^{j}$ is the HDP diagnosis rate for rurality status j (a = urban, b = rural).

$\:{\beta\:}^{j}$ is a vector of coefficient estimates for rurality status, j (urban or rural).

$\:{X}^{j}$ is a row vector of average values of the independent variables for rurality status, j (urban or rural).

Second, following Fairlie (27), the difference between urban and rural pregnant women’s HDP status can be decomposed as:

1 $$ \begin{aligned} \:\overline{{Y^{a} }} \: - \overline{{\:Y^{b} }} = & \left[ {\sum {\:_{{i = 1}}^{{N^{a} }} } \frac{{F\left( {X_{i}^{a} \beta \:^{b} } \right)}}{{N^{a} }} - \sum {\:_{{i = 1}}^{{N^{b} }} } \frac{{F\left( {X_{i}^{b} \beta \:^{b} } \right)}}{{N^{b} }}} \right] \\ & + \left[ {\sum {\:_{{i = 1}}^{{N^{a} }} } \frac{{F\left( {X_{i}^{a} \beta \:^{a} } \right)}}{{N^{a} }} - \sum {\:_{{i = 1}}^{{N^{a} }} } \frac{{F\left( {X_{i}^{a} \beta \:^{b} } \right)}}{{N^{a} }}} \right] \\ \end{aligned} $$

In the above equation:

$\:\overline{{Y}^{j}}$ is the average probability of the binary outcome, HDP status, for rurality status, j (a = urban, b = rural), $\:\overline{{Y}^{a}}\:$-$\:\overline{{Y}^{b}}$ represents the total variation in HDP status due to group (urban versus rural) differences; $\:{N}^{a}$and $\:{N}^{b}$ are the sample sizes of the urban and rural populations.

In Eq. (1), the first term is the explained segment - that is, the part of the urban-rural gap in HDP rate that is due to group differences in distributions of the independent variables, X. The second half is the unexplained segment - the portion of the rurality gap due to group differences in unmeasurable or unobserved endowments (Fairlie, 1999; Fairlie & Robb, 2007; Jann, 2006). To ensure the stability of the results, we used the software to repeat the calculation of the decomposition model 1000 times.

## Results

### Descriptive Statistics

Chi-square tests in Table 1 revealed significant differences in the distribution of several variables across rural and urban pregnant women with HDP: MDP (Panel A), highest level of education, race/ethnicity, insurance type, age (categories), prenatal care, chronic hypertension, and diabetes (Panel B). T-tests also revealed statistically significant differences in means across rural and urban areas for maternal age years) and gestational age (weeks). Across rural and urban pregnant women with HDP, there were no significant differences in the distribution of PMAD (Panel A), gestational diabetes or obesity(Panel B).

**Table 1:** Summary statistics of urban and rural pregnant women with HDP in SC: 1999–2017

	Urban pregnant women with HDP (*N* = 13,156)	Rural pregnant women with HDP (*N* = 3,009)	*P*-value from Chi-Squared test
*Panel A: Mental health diagnoses*			
Perinatal mood and anxiety disorders (PMAD)	1,061 (8.06%)	253 (8.16%)	0.855
Maternal mental disorders of pregnancy (MDP)	748 (5.69%)	120 (3.87%)	0.000
*Panel B: Demographic characteristics*			
***Highest level of education***			0.000
High school	4,480 (34.29%)	1,365 (44.46%)	
Some college	4,715 (36.09%)	1,147 (37.36%)	
College degree	2,622 (20.02%)	390 (12.70%)	
More than college degree	1,247 (9.55%)	168 (5.47%)	
***Race***			0.000
White	7,995 (60.77%)	1,616 (52.15%)	
Black or African American	4,241 (32.24%)	1,327 (42.82%)	
Hispanic	383 (2.91%)	60 (1.94%)	
Other	537 (4.08%)	96 (3.10%)	
***Age (categorical)***			0.000
19 and under	1,221 (9.28%)	379 (12.23%)	
20–29	7,793 (59.24%)	1,985 (64.05%)	
30–39	3,822 (29.05%)	672 (21.68%)	
40 and above	320 (2.43%)	63 (2.03%)	
***Insurance type***			0.000
Medicare/Medicaid	5,274 (40.10%)	1,549 (49.98%)	
Private insurance	7,058 (53.66%)	1,384 (44.66%)	
Veteran’s administration	517 (3.93%)	114 (3.68%)	
Self-pay/No insurance	304 (2.31%)	52 (1.68%)	
***Mother’s prenatal care adequacy (Kotelchuck)***			0.001
Inadequate	1,763 (13.58)	427 (14.03%)	
Intermediate	543 (4.18%)	162 (5.32%)	
Adequate	2,781 (21.42%)	572 (18.80%)	
Adequate plus	7,895 (60.81%)	1,882 (61.85%)	
***Diabetes***			0.000
Yes	507 (3.85%)	176 (5.68%)	
***Chronic hypertension***			0.000
Yes	3,039 (23.10%)	808 (26.07%)	
***Gestational age (weeks)***			***P*** **-value from T- test of difference in means**
Mean	37.571	37.291	0.000
Standard deviation	2.856	3.144	

The table reports the p-value from Chi-squared tests for the categorical variables and from T-tests of differences in means for continuous variables to determine any relationship between the variables and the rurality status

### Logistic Model Results

Table 2 presents the logistic model results for urban pregnant women. The results indicate that the odds of being diagnosed with HDP decrease with greater maternal education (College, OR = 0.811; More than College, OR = 0.715); if the mother received intermediate (OR = 0.714) or adequate (OR = 0.817) prenatal care compared to inadequate prenatal care; if the mother had a longer gestation period (OR = 0.931); if the mother had VA insurance compared to having public insurance (OR = 0.863); and if the mother identified as Hispanic or of Other race category relative to identifying as White (Hispanic, OR = 0.758; Other, OR = 0.666). The odds of being diagnosed with HDP increased with a PMAD (OR = 1.239) or MDP diagnosis (OR = 1.292); identifying as Black (OR = 1.121 for the PMAD model and 1.126 for the MDP model); if the mother had private insurance relative to public insurance (OR = 1.044 for MDP model); the mother being older (OR = 1.024 for PMAD model and OR = 1.023 for MDP model); if the mother had diabetes (OR = 1.510 for PMAD model and OR = 1.845 for MDP model), chronic hypertension (OR = 26.10 for both models) or gestational diabetes (OR = 1.822 for both models); and was obese (OR = 1.853 for PMAD model and OR = 1.852 for MDP model).

**Table 2:** Logistic regression results for HDP status among urban SC pregnant women who have given birth only once: 1999–2017

	Only PMAD model	Only MDP model
*Mental health experiences*		
PMAD	1.239*** (0.065)	
MDP		1.291*** (0.059)
*Maternal covariates*		
*Education* *Reference: high school*		
Some college	0.977 (0.025)	0.981 (0.025)
College degree	0.811*** (0.027)	0.816*** (0.027)
More than college degree	0.715*** (0.030)	0.719*** (0.030)
*Insurance type* *Reference: Medicaid/Medicare*		
Self-pay/indigent/none	0.904 (0.062)	0.906 (0.062)
Private	1.039 (0.027)	1.044* (0.027)
Veterans administration	0.863*** (0.044)	0.868*** (0.044)
Age (years)	1.024*** (0.002)	1.023*** (0.002)
Black or African American (1 = Yes)	1.121*** (0.027)	1.126*** (0.027)
Hispanic (1 = Yes)	0.758*** (0.043)	0.763*** (0.044)
Other race (1 = Yes)	0.666*** (0.032)	0.670*** (0.032)
*Pregnancy related care and health*		
*Prenatal care* *Reference: inadequate prenatal care*		
Intermediate prenatal care	0.714*** (0.037)	0.715*** (0.037)
Adequate prenatal care	0.817*** (0.027)	0.819*** (0.028)
Adequate plus prenatal care	1.209*** (0.036)	1.212*** (0.036)
Gestation age (weeks)	0.931*** (0.003)	0.931*** (0.003)
Gestational diabetes (1 = yes)	1.822*** (0.078)	1.822*** (0.078)
*Other maternal conditions*		
Chronic hypertension (1 = Yes)	26.10*** (0.959)	26.10*** (0.959)
Diabetes (1 = Yes)	1.510*** (0.138)	1.845*** (0.142)
Obese (1 = Yes)	1.853*** (0.066)	1.852*** (0.065)
Constant	0.354*** (0.053)	0.348*** (0.052)
Time dummies	Yes	Yes
Observations	225,408	225,408

The table reports the odds ratios from the logistic regressions of HDP status of urban pregnant women on the listed independent variables. Refer to text for definitions of covariates. Robust standard errors are included in parentheses. Statistical significance is denoted as follows: *** *p* < 0.01, ** *p* < 0.05, * *p* < 0.10

Table 3 presents the logistic model results for rural pregnant women. The results indicate no significant association between PMAD or MDP and the odds of being diagnosed with HDP. Comorbidities, age, gestational diabetes, and gestational age were similar to the urban sample in their odds of raising the HDP diagnoses. Most of the other maternal characteristics were not statistically significant.

**Table 3:** Logistic regression results for HDP status among rural SC pregnant women who have given birth only once: 1999–2017

	Only PMAD model	Only MDP model
*Mental health experiences*		
PMAD	1.039 (0.151)	
MDP		1.108 (0.125)
*Maternal covariates*		
*Education* *Reference: high school*		
Some college	0.982 (0.048)	0.984 (0.048)
College degree	0.871* (0.066)	0.872* (0.066)
More than college degree	0.720*** (0.074)	0.721*** (0.074)
*Insurance type* *Reference: medicaid/medicare*		
Self-pay/indigent/none	1.096 (0.183)	1.099 (0.183)
Private	0.998 (0.050)	1.000 (0.050)
Veterans administration	0.613*** (0.065)	0.614*** (0.065)
Age (years)	1.035*** (0.005)	1.035*** (0.005)
*Race/ethnicity* *Reference: non-hispanic white*		
Black or African American (1 = Yes)	1.014 (0.046)	1.017 (0.046)
Hispanic (1 = Yes)	0.775* (0.111)	0.777* (0.112)
Other race (1 = Yes)	0.653*** (0.079)	0.655*** (0.079)
*Pregnancy related care and health*		
*Prenatal care* *Reference: inadequate prenatal care*		
Intermediate prenatal care	0.777** (0.078)	0.778** (0.078)
Adequate prenatal care	0.776*** (0.056)	0.777*** (0.056)
Adequate plus prenatal care	1.033 (0.065)	1.034 (0.065)
Gestation age (weeks)	0.933*** (0.006)	0.933*** (0.006)
Gestational diabetes (1 = yes)	1.874*** (0.172)	1.873*** (0.172)
*Other maternal conditions*		
Chronic hypertension (1 = Yes)	26.46*** (1.935)	26.45*** (1.934)
Diabetes (1 = Yes)	2.351*** (0.463)	2.440*** (0.334)
Obese (1 = Yes)	1.607*** (0.127)	1.605*** (0.127)
Constant	0.305*** (0.091)	0.301*** (0.090)
Time dummies	Yes	Yes
Observations	49,591	49,591

The table reports the odds ratios from the logistic regressions of HDP status of rural pregnant women on the listed independent variables. Refer to text for definitions of covariates. Robust standard errors are included in parentheses. Statistical significance is denoted as follows: *** *p* < 0.01, ** *p* < 0.05, * *p* < 0.10

### Fairlie Decomposition Results

**Fig. 1:**
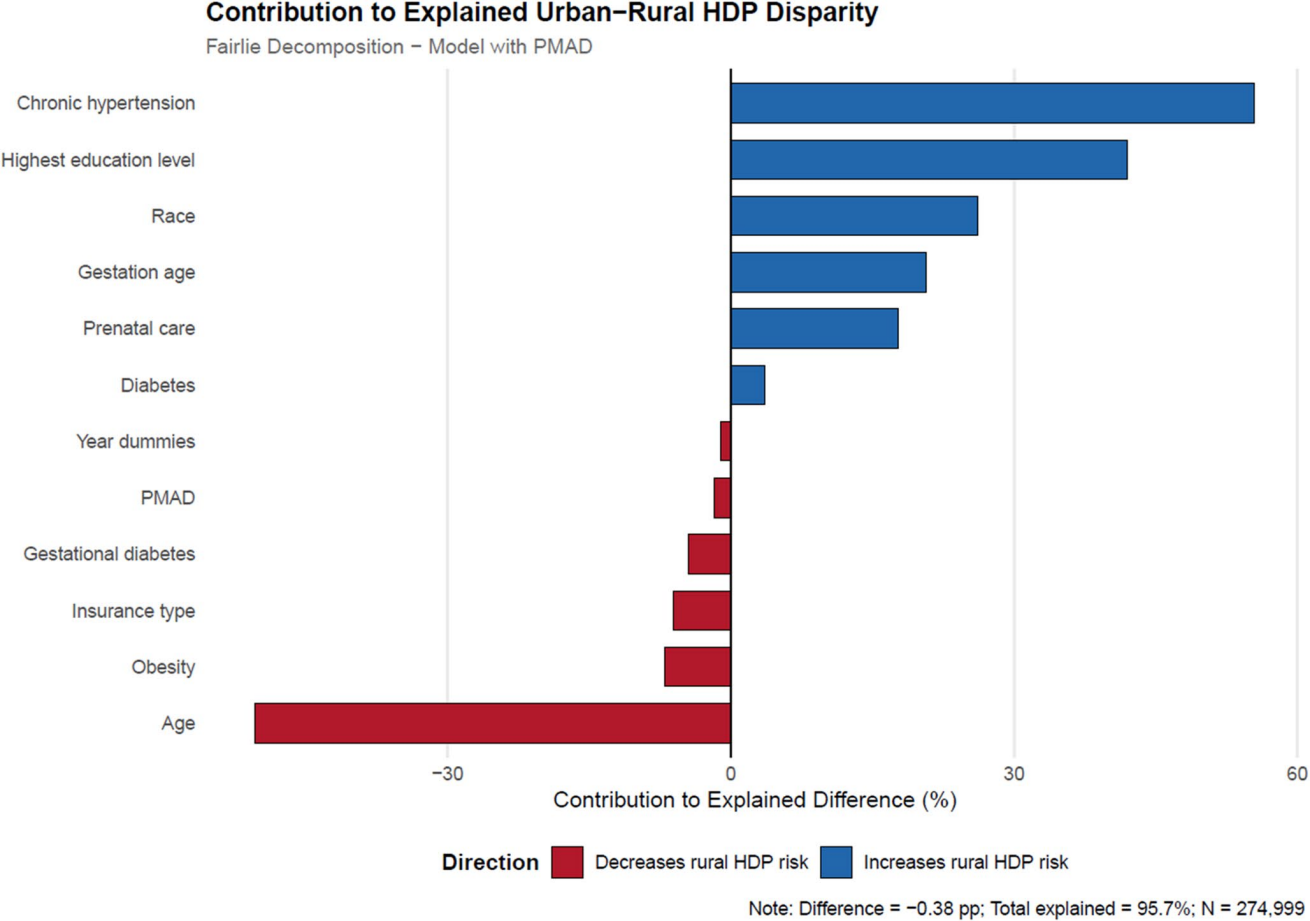
Variable contributions to the explained component of urban-rural HDP disparity, including PMAD. *See text for details. Refer to the Fairlie Decomposition Results section for thepercentage contributions of each of the factors to the rural-urban disparity in HDP diagnosis,including PMAD.

**Fig. 2:**
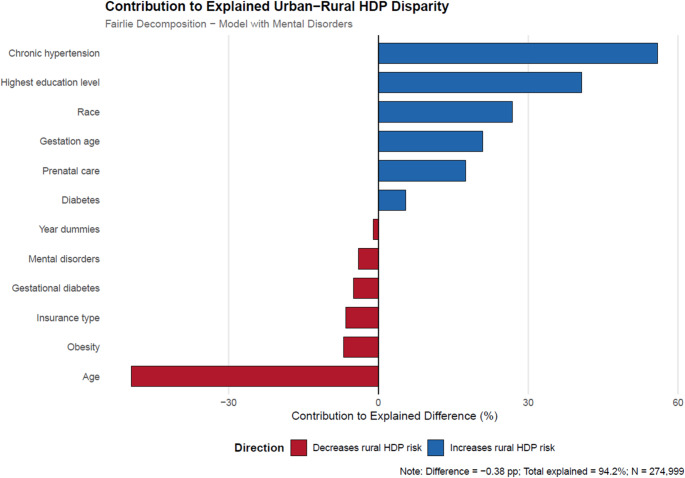
Variable contributions to the explained component of urban-rural HDP disparity, including MDP. *See text for details. Refer to the Fairlie Decomposition Results section for thepercentage contributions of each of the factors to the urban-rural disparity in HDP diagnosis,including MDP.

The decomposition results in Tables 4 and 5 estimate how much of the urban-rural HDP gap is due to differences in characteristics between urban and rural pregnant women, including mental health diagnoses such as PMAD (Table 4; Fig. 1) and MDP (Table 5; Fig. 2). The first two rows of both tables show the rate of HDP in rural areas (6.1%) and urban areas (5.7%), forming an urban-rural gap of −0.376% points.

**Table 4:** Fairlie decomposition of HDP diagnosis disparity between urban and rural SC pregnant women: only PMAD

Terms of decomposition	HDP
Urban HDP rate	0.05737596
Rural HDP rate	0.06114013
Difference (urban-rural)	−0.00376417
Explained (%)	−0.00360128 (96%)
Unexplained (%)	−0.00016289 (4%)

*See text in Method and Fairlie Decomposition Results sections for the explained and unexplained percentages.

**Table 5:** Fairlie decomposition of HDP diagnosis disparity between rural and urban SC pregnant women: only MDP

Terms of decomposition	HDP
Urban HDP rate	0.05737596
Rural HDP rate	0.06114013
Difference (urban-rural)	−0.00376417
Explained (%)	−0.00360128 (94%)
Unexplained (%)	−0.00016289 (6%)

*See text in Method and Fairlie Decomposition Results sections for the explained and unexplianed percentages.

The decomposition results in Table 4 reveal that observed factors caused 96% of the HDP diagnosis differences, whereas 4% of the differences were caused by region (rural and urban) and unobserved factors. Therefore, this decomposition model controlling for PMAD explained 96% of the HDP diagnosis differences between urban and rural pregnant women in South Carolina. The decomposition reveals that one of the most important contributing factors is whether the mother had chronic hypertension. Relatively high levels of chronic hypertension among rural pregnant women with HDP explain − 0.21% points (or 55%) of why HDP rates are higher for this group. Another critical factor is maternal education which explains 42% of why HDP rates are higher for rural pregnant women. The lower education levels of rural pregnant women contribute to why they have higher HDP rates than their urban counterparts.

Lower maternal age favors rural pregnant women, as evidenced by the negative contribution estimate (−50%). About 76% of rural pregnant women are 39 or younger, while about 68% of urban pregnant women are in that age range, as shown in Table 1. In addition, differences in maternal comorbidity are favorable for rural pregnant women relative to urban pregnant women, as evidenced by the negative contribution estimates. The contribution estimate of −7% for maternal obesity indicates that rural pregnant women have lower obesity rates (also shown in Table 2) and being a risk factor for HDP (Egeland et al., 2016), is a favorable factor for rural pregnant women relative to urban pregnant women. Similarly, the contribution estimate of −4% for gestational diabetes indicates that lower rates for rural pregnant women (see Table 1) relative to urban pregnant women is yet another favorable factor for rural pregnant women relative to urban pregnant women. The estimated contribution of − 6% for pregnant women’s insurance indicates that rural pregnant women are more likely to have some kind of insurance, and this characteristic is favorable for them compared to urban pregnant women. Table 1 shows that only 1.68% of rural pregnant women have no insurance or self-pay for care relative to 2.31% of urban pregnant women. As for a PMAD diagnosis, a contribution estimate of −2% shows that it’s a small favorable characteristic for rural pregnant women, although it’s neither substantial nor statistically significant at *p* < 0.01.

The decomposition results in Table 5 accounting for MDP diagnosis are very similar to the results in Table 4 focusing on PMAD diagnosis. The results show that observed factors caused 94% of the HDP diagnosis differences, whereas 6% of the differences were caused by region (rural and urban) and unobserved factors. Therefore, this decomposition model controlling for MDP explained 96% of the HDP diagnosis differences between urban and rural pregnant women in South Carolina. The decomposition reveals that one of the most important contributing factors is whether the pregnant woman had chronic hypertension. Relatively high levels of chronic hypertension among rural pregnant women with HDP explain − 0.21% points (or 56%) of why HDP rates are higher for this group. Another important factor is maternal education which explains 41% of why HDP rates are higher for rural pregnant women. The lower education levels of rural pregnant women contribute to why they have higher HDP rates than their urban counterparts.

Lower maternal age is favorable to rural pregnant women, as evidenced by the negative contribution estimate (−49%). About 76% of rural pregnant women are 39 or younger, while about 68% of urban pregnant women are in that age range as shown in Table 1. In addition, differences in maternal comorbidity are favorable for rural pregnant women relative to urban pregnant women, as evidenced by the negative contribution estimates. The contribution estimate of −7% for maternal obesity indicates that rural pregnant women have lower obesity rates (also shown in Table 1) and being a risk factor for HDP (30), is a favorable factor for rural pregnant women relative to urban pregnant women. Similarly, the contribution estimate of −5% for gestational diabetes indicates that lower rates for rural pregnant women (see Table 1) relative to urban pregnant women is yet another favorable factor for rural pregnant women relative to urban pregnant women. The contribution estimate of − 7% for the pregnant woman’s insurance indicates that rural pregnant women are more likely to have some kind of insurance and this characteristic is favorable for them relative to urban pregnant women. As for a MDP diagnosis, a contribution estimate of −4% shows that it’s a favorable characteristic for rural pregnant women relative to urban pregnant women since they have a lower rate of MDP diagnosis (3.87% for rural versus 5.69% for urban in Table 1) as well as being a significant predictor of HDP diagnosis in the urban sample (Table 2) and not for the rural sample (Table 3).

## Discussion

The aim of this study is to examine the variation in HDP diagnosis based on the mother’s rurality status as well as the explanatory role of clinical factors such as diagnosed mental health and comorbidities, and sociodemographic factors, like age, education, insurance provider, and race/ethnicity. In general, we found a higher rate of HDP in rural areas with comorbidity, education, race, prenatal care, and maternal age as significant factors in explaining urban-rural differences among HDP diagnoses. Our results confirm prior analyses, which also found a higher incidence of HDP in rural locations (Cameron et al., 2020, 2022). Our results provide new evidence on the potential contribution of maternal mental health disorders such as PMAD and MDP and HDP presentation at delivery for urban pregnant women and indicate that urban-rural differences in such diagnosed maternal mental health conditions actually reduce the urban-rural gap in the HDP rate.

The study’s findings align with existing literature that emphasizes the multifaceted nature of HDP risk factors. Findings confirm the protective effect of adequate prenatal care and longer gestation periods, emphasizing the importance of healthcare access throughout the perinatal period for maternal and child well-being (Cameron et al., 2022; Cameron et al., 2020).

Maternal mental health conditions are increasing across the US (Pino et al., 2019). Although limited work has shown that pregnant women with anxiety are at higher risk for HDP (Raina et al., 2021); our work extends this finding by demonstrating that PMAD and MDP contribute to rural-urban disparities in HDP, effectively reducing the urban-rural gap in HDP rates. Prior research has shown that a PMAD diagnosis was associated with an increased risk for HDP (Runkle et al., 2023). Within our sample, both PMAD and MDP were associated with a higher likelihood of HDP among urban pregnant women, but these factors were not statistically significant in the rural subsample. Nationally, other work has found higher perinatal anxiety among older women, white, and from higher income quartiles, and that perinatal anxiety increases the risk of HDP (Pino et al., 2019; Raina et al., 2021). We add to this evidence by finding that while MDP seems to decrease the urban-rural disparity, the most established risk factors (e.g., mother’s race, mother’s education, whether the mother had chronic hypertension, gestation age in weeks, and prenatal care) still account for urban-rural differences in HDP.

Policymakers should consider the multifaceted nature of maternal health by incorporating mental health components into strategies for addressing HDP. Targeted interventions, informed by these findings, could potentially reduce the burden of HDP and mitigate urban-rural disparities in maternal health outcomes. Routine screenings for PMAD and MDP, coupled with improved access to mental health support services, could potentially alleviate the burden of HDP, especially among urban populations where these conditions are more prevalent.

## Conclusion

This paper studies the urban-rural disparity in HDP, emphasizing the often overlooked role of maternal mental health conditions. Using the Fairlie decomposition approach and hospital discharge data from South Carolina, we find that MDP explains about − 4% of the urban-rural disparity in HDP diagnosis, indicating it’s a favorable characteristic for rural pregnant women relative to urban pregnant women since they have a lower rate of MDP diagnosis. Other contributing factors include higher maternal age, gestational diabetes, comorbidities, and lower education levels. Although this study does not establish causation, it is among the first to shed light on the importance of maternal mental health conditions in understanding the urban-rural gap in maternal health outcomes.

## Data Availability

Data for this project are unsuitable to post publicly owing to a data use agreement with the state of South Carolina’s Revenue and Fiscal Affairs Office Health and Demographics division.

## Code Availability

Researchers used the statistical software Stata18 (version 18) to run the analyses.
